# *Glenea coomani* Pic, 1926 and its related species of South China with description of a new species

**DOI:** 10.3897/zookeys.153.2106

**Published:** 2011-12-09

**Authors:** Meiying Lin, Xingke Yang

**Affiliations:** 1 Key Laboratory of Zoological Systematics and Evolution, Institute of Zoology, Chinese Academy of Sciences, Beichen West Road, Chaoyang Dist., Beijing, 100101, China

**Keywords:** *Glenea*, new species, taxonomy, distribution, Oriental region

## Abstract

*Glenea coomani* Pic, 1926 distributed in Vietnam, Laos and China is redescribed, and its sibling species, *Glenea neohumerosa* **sp. n.** is described from China (Guangxi, Hainan and Fujian) and North Vietnam. They are separated from each otherby differences in genitalia, and apical teeth and maculae of elytra. Another four related species and one subspecies are illustrated with short notes and new localities, and the lectotype and paralectotype of *Glenea tonkinea* Aurivillius, 1925 are designated. A key to the related species is presented.

## Introduction

*Glenea coomani* Pic, 1926 was originally described from North Vietnam, and *Glenea humerosa* Gressitt, 1940, described from Hainan Island, China had been synonymized with it by Breuning (1956). In the course of our study of saperdine beetles from South China, we confirmedtheir conspecific status based on the study of the type material. However, we surprisingly found another species, which had been identified as *Glenea coomani* or *Glenea humerosa* by predecessors. This new species is distinguishable from *Glenea coomani* by having different elytral maculae, longer elytral apical teeth, and differing structure of male genitalia. Therefore, we describe *Glenea neohumerosa* sp. n., and compare it with *Glenea coomani* which is redescribed. We show the habitus of similar species which are compared with short notes and new localities. The following species are mentioned and keyed: *Glenea coomani* Pic, 1926, *Glenea neohumerosa* sp. n., *Glenea lacteomaculata* Schwarzer, 1925, *Glenea lacteomaculata quadriguttata* Pic, 1926, *Glenea laodice* Thomson, 1879, *Glenea subalcyone* Breuning, 1964, *Glenea tonkinea* Aurivillius, 1925.

## Materials and methods

Types and other material studied are deposited in the following institutions or private collections:

BM	Bishop Museum, Honolulu, USA

CAS	California Academy of Sciences, San Francisco, USA

CAU	China Agricultural University, Beijing, China

CBWX	Collection of Wenxuan Bi, Shanghai, China

CCCC	Collection of Chang-chin Chen, Tianjin, China

CJM	Collection of Ming Jin, Shanghai, China

CWD	Collection of Dong Wen, Qingdao, Shandong, China

IZAS	Institute of Zoology, Chinese Academy of Sciences, Beijing, China

MNHN	Muséum National d'Histoire Naturelle, Paris, France

MHNL	Muséum d'Histoire Naturelle, Lyon, France

NHRS	Swedish Museum of Natural History, Stockholm, Sweden (= Naturhistoriska Riksmuseet Stockholm)

NMB	Naturhistorisches Museum, Basel, Switzerland (including ex Museum G. Frey, Tutzing)

SHEM	Shanghai Entomology Museum, Shanghai, China

SYSU	Sun-Yatsen University, Guangzhou, China

## Results

### Glenea coomani

Pic

http://species-id.net/wiki/Glenea_coomani

Figs 1 11

Glenea coomani  Pic, 1926: 21(Tonkin). [MNHN]
Glenea humerosa  Gressitt, 1940: 206, pl. 6, fig. 4 (Hainan). [CAS, syn. by Breuning 1956]
Glenea  (s. str.) *coomani*; Rondon and Breuning 1971: 537 (Laos).
Glenea coomani ; Breuning 1956: 744; Hua 2002: 210.

#### Redescription.

**Figures 1–6.**
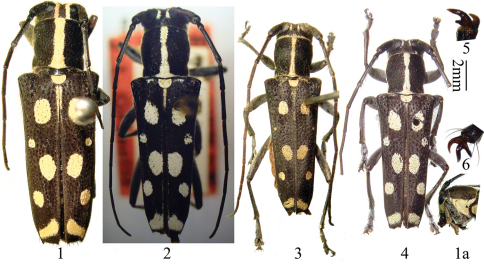
Habitus, *Glenea coomani* Pic. **1** holotype, female, from Tonkin, Vietnam **1a** showing lateral pubescent stripe on prothorax **2** holotype of *Glenea humerosa* Gressitt, male, from Hainan, China (picture from Carolus Holzschuh) **3** male, from Yunnan, China **4** male, from Hainan, China. Scale 2 mm. **1a, 5–6** not to scale **5** showing claw of front tarsus **6** showing claw of mid tarsus.

Male (Figs 2–6): length: 11.5–14.2 mm, humeral width: 3.5–4.5 mm. Female (Fig. 1): length: 14.0–16.2 mm, humeral width: 4.5–5.4 mm. Body black, in part provided with white to pale yellow pubescent maculae. Head with white to pale yellow maculae on genae, borders of eyes, temple and two parallel stripes between upper eye lobes; antennae black, with thin, whitish pubescence on inner sides of first three segments and base of fourth segment, and scattered with short, black bristles on undersides of first seven segments. Prothorax with a medial white to pale yellow stripe, and each side white to pale yellow (Fig. 1a). Scutellum white to pale yellow. Elytra with suture narrowly white to pale yellow near base; each disc with 5 white to pale yellow maculae: a large oval spot close to suture at the end of basal 1/4; a smallest spot near the middle, far from suture; the third one large, oval, close to suture just behind the middle; the fourth medium in size, round, far from suture; an oblique transverse band just before apex. Ventral surface covered with dense white to pale yellow pubescence, thinly so along middle. Legs black, thinly pubescent. Pronotum and elytral bases with sparse, erect, black bristles.

Head hardly broader than prothorax, deeply, and in part densely punctured, feebly concave at vertex. Eyes deeply emarginate, inferior eye lobes subequal to (female), or 2 times as high as (male) genae below it, width much less than half of front. Antennae longer than body; scape slightly thickened apical without cicatrix nor a ridge; antennomere ratio (male): 12 : 3 : 21 : 16 : 16 : 15 : 14 : 13 : 13 : 12 : 13; (female): 15 : 4 : 24 : 17 : 17 : 16 : 15 : 14 : 14 : 13 : 13. Prothorax almost as broad as long (male) or broader than long (female), swollen laterally before middle; disc convex and somewhat deeply and closely punctured. Elytra prominently angulate at humeri, slightly narrowed apically; each with 2 humeral longitudinal ridges beginning after humeri and reaching near the apex, truncated apically, with short and small teeth at the suture and the outer angle, surface with coarse and irregular punctures. Legs stout, middle tibiae grooved, hind femur reaching fourth abdominal segment, first hind tarsal segment longer than (male), or nearly as long as (female) following two segments combined. Male claws: the anterior claws of the fore and mid tarsi are toothed at the base, but the tooth in the fore tarsus is very small (Fig. 5), the tooth in the mid tarsus is long (Fig. 6, almost same size of the normal claw). Female claws simple.

**Figures 7–11.**
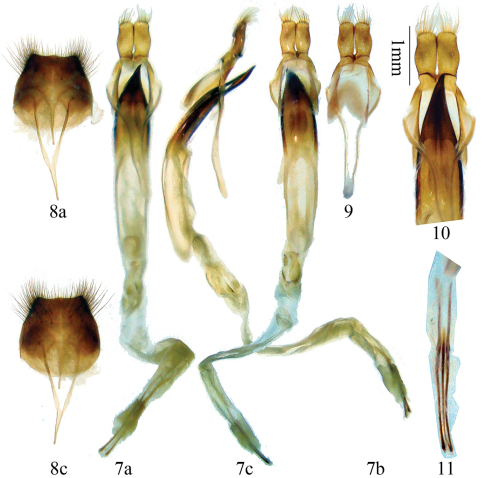
Terminalia of *Glenea coomani* Pic **7** male genitalia **8** tergite VIII and sternite VIII & IX **a** ventral view **b** lateral view **c** dorsal view **9** tegmen, showing the basal piece. Scale 1 mm. **10–11** not to scale **10** showing ridges in base of lateral lobes and apex of ventral plate of median lobe **11** showing three rods of endophallus.

Male genitalia (Figs 7–11): Tegmen length about 3.0 mm; lateral lobes stout, each about 0.6 mm long and 0.3 mm wide, with fine haired ridge at the base (in ventral view), apex nearly truncated and with fine setae which are shorter than lateral lobes; ringed part elbowed in the widest portion, converging; basal piece bifurcated distally (Fig. 9); median lobe plus median struts moderately curved, a little longer than tegmen (6:5); the median struts about one half of the whole length of median lobe; dorsal plate shorter than ventral plate; apex of ventral plate narrowly pointed, with sharp apex which is always curved to right side (in ventral view, Fig. 10); median foramen elongated triangular, with a small projection in lateral view; internal sac about 3 times as long as median lobe plus median struts, with 4 pieces of basal armature, 2 bands of supporting armature and 3 rods; the two longer rods each about 1.6 mm, shorter than tegmen, the short middle rod about 1.1 mm long. Ejaculatory duct single. Tergite VIII (Fig. 8c) broader than long, apex truncated with middle slightly projected, setae near lateral corner dense and long, and sparse and short around middle.

#### Diagnosis.

Differs from *Glenea lacteomaculata* Schwarzer, 1925 (Fig. 24), *Glenea lacteomaculata quadriguttata* Pic, 1926 (Figs 25–26) and *Glenea tonkinea* Aurivillius, 1925 (Figs 30–33) in elytron having only one big oval macula at basal fourth, instead of two small spots, the second macula smallest instead of the first one. Differs from *Glenea laodice* Thomson, 1879 (Fig. 27) and *Glenea subalcyone* Breuning, 1964 (Figs 28–29) in elytron without long and sharp tooth at the outer angle, having only one big oval macula at basal fourth, instead of two spots.

#### Remarks.

Based on the study on the types and material from type localities, we agreed with Breuning (1956) that *Glenea humerosa* Gressitt, 1940 is conspecific with *Glenea coomani* Pic, 1926. Though the holotype of *Glenea humerosa* Gressitt (Fig. 2) has the middle oval pubescent spot reaching suture and seems to be different from the type of *Glenea coomani* Pic (Fig. 1), the male (Fig. 4) also from Hainan Island looks no different from those from Tonkin. Nevertheless, such pubescent markings are quite variable in shape and size within same species.

#### Distribution.

China: Hainan, Yunnan (new province record); Vietnam, Laos.

#### Type specimen examined.

Holotype of *Glenea coomani* Pic, female, Tonkin (MNHN). Holotype of *Glenea humerosa* Gressitt, male, Ta-hian, foot of Five Finger Moutains, southcentral Hainan, 1935.VI.18, leg. Gressitt (CAS) [by original description and pictures].

#### Other specimens examined.

**China: Yunnan:** 1 male, Cheli to Damenglong, alt. 600 m, 1957.IV.22, leg. Dahua Liu (IZAS). **Hainan:** 1 male, Ledong, 1984.VIII.26, leg. Zhiqing Chen (IZAS). **Vietnam:** 1 male, Tonkin, Hoa-Binh (MNHN); 1 female, same data but (NMB, ex Coll. Frey); 1 male, Tonkin, Hoa-Binh, leg. A. de Cooman (IZAS); 1 female, same data but (NMB, ex Coll. Frey). **Laos:** 2 males, Ban Van Heue, 20 km E. of Phou-kow kuei, 1965.V.1–15, leg. J. A. Rondon (BM); 18 males 18 females, Phontiou, 1965.V.15 (MNHN, ex Coll. J. Rondon, 1967).

### Glenea neohumerosa sp. n.

urn:lsid:zoobank.org:act:9C4BC446-D2E5-4E4E-9BDD-F66245CC93C5

http://species-id.net/wiki/Glenea_neohumerosa

Figs 12 23 

#### Description.

**Figures 12–18.**
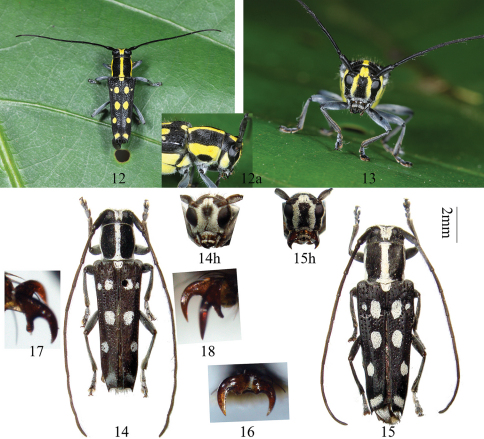
Habitus, *Glenea neohumerosa* sp. n. **12–13** paratype, female, from Hainan, China, showing yellow coloration of fresh material (not to scale) (taken by Wenxuan Bi in June, 2011) **14** holotype, male, from Guangxi, China **14h** head of male, frontal view **15** female, from Hainan, China **15h** head of female, frontal view. Scale 2 mm **16** simple claw, showing claw of front tarsus of male. **17–18** showing claw of mid tarsus of male (not to scale).

Male (Figs 14, 14h, 16–18): length: 8.4–10.8 mm, humeral width: 2.3–3.0 mm. Female (Figs 12–13, 15, 15h): length:10.5–13.0 mm, humeral width: 3.2–3.9 mm. Body black, in part provided with thick, white (dry and old specimens, Figs 14–15) to yellow (alive or fresh specimens, Figs 12–13) pubescent maculae. Head black, frons with two white or yellow stripes (almost fused in male, Fig. 14h) from inner side of antennae insertions along eyes and genae to clypeus (Fig. 15h), temple white or yellow (Fig. 12a), vertex with two parallel stripes (usually fused) between upper eye lobes; antennae black, scattered with short, black bristles on undersides of first seven segments. Prothorax with a medial white or yellow stripe, each side white or yellow except a transverse black vitta (Fig. 12a). Scutellum white or yellow. Elytra without surural stripes, each disc with 5 white or yellow maculae: two spots at basal 1/4, the one near suture much bigger than the one near margin; a moderate sized oval spot at middle, near suture; the fourth one smaller than middle one, closer to lateral margin than to suture, at the centre of apical half; an oblique transverse band just before apex. Ventral surface covered with dense white or yellow pubescence, thinly so along middle. Legs black, thinly pubescent. Pronotum and elytral bases with sparse, erect, black bristles.

Head hardly broader than prothorax, deeply, and in part closely punctured, feebly concave at vertex. Eyes deeply emarginate, inferior eye lobes subequal to (female) or 2 times as high as (male) genae below it, width much less than half of frons. Antennae longer than body, male longer than female; scape thicknened apical without cicatrix not a ridge; antennomere ratio (male): 12 : 3 : 18 : 15 : 14 : 13 : 13 : 12 : 12 : 11 : 12; (female): 13 : 3 : 21 : 17 : 16 : 15 : 14 : 14 : 13 : 12 : 13. Prothorax almost as broad as long (male) or broader than long (female), swollen laterally before middle, disc convex and somewhat deeply and closely punctured. Elytra rounded at humeri, slightly narrowed apically, each with 2 humeral longitudinal ridges beginning after humeri and reaching close to apex, truncated apically, with short and small teeth at the suture, long and sharp spine at the outer angle, surface with coarse and irregular punctures. Legs stout, middle tibiae grooved, hind femur reaching middle to apex of third abdominal segment, first hind tarsal segment longer than (male), or nearly as long as (female) following two segments combined. Male claws: the anterior claws of the mid tarsi with a short (half of the normal claw) tooth (Figs 17–18), fore and hind tarsi with simple claws (Fig. 16). Female claws simple.

**Figures 19–23.**
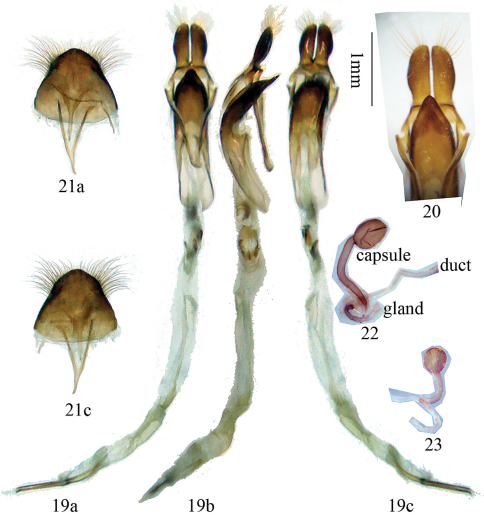
Terminalia of *Glenea neohumerosa* sp. n. **19** male genitalia. **20** showing ridges in base of lateral lobes and apex of ventral plate of median lobe (not to scale) **21** tergite VIII and sternite VIII & IX **a** ventral view **b** lateral view **c** dorsal view **22–23** female genitalia **22** spermathecal capsule distorted (not to scale). Scale 1 mm.

Male genitalia (Figs 19–21): Tegmen length about 1.9 mm; lateral lobes slender, each about 0.6 mm long and 0.2 mm wide, with finely haired ridge at the base (in ventral view, Fig. 20), apex obliquely rounded and with fine setae which are shorter than lateral lobes; ringed part elbowed in the widest portion, converging; basal piece bifurcated distally; median lobe plus median struts moderately curved, subequal to tegmen in length; the median struts about one half of the whole length of median lobe; dorsal plate shorter than ventral plate; apex of ventral plage (Fig. 20) pointed, apex not so sharp and not curved to right side; median foramen elongated with a projection in lateral view (Fig. 19b); internal sac about 3 times as long as median lobe plus median struts, with 4 pieces of basal armature, 2 bands of supporting armature and 3 rods; the two longer rods each about 1.2 mm, shorter than tegmen, the short middle rod about 0.7 mm long. Ejaculatory duct single. Tergite VIII (Fig. 21c) longer than broad, apex rounded, setae near lateral corner dense and long, and sparse and short around middle.

Female genitalia (Figs 22–23): spermathecal gland located at the base of spermathecal capsule. Spermathecal capsule with a curved basal stalk and a rounded apical orb, stalk more than twice the length of capsule.

#### Diagnosis.

Differs from long spine elytron (Fig. 35) species *Glenea laodice* Thomson, 1879 (Fig. 27) and *Glenea subalcyone* Breuning, 1964 (Figs 28–29) in elytron having five white or yellow maculae instead of six, and their positions different. Differs from *Glenea coomani* and other short tooth elytron (Fig. 34) species in elytral apex having a long and sharp spine at the outer angle. Differs from *Glenea coomani* also in male terminalia: tergite VIII with apex rounded instead of truncated; lateral lobes of tegmen slender, the length ratio of lateral lobes to tegmen much bigger; apex of ventral plate not so sharp and not curved to right side.

#### Etymology.

Named derived on similarity to and misidentification as *Glenea humerosa* by Gressitt and Hua (based on material deposited in SYSU and IZAS).

#### Remarks.

The yellow color of the pubescence turns into white when the specimens are dried.

#### Distribution.

China: Guangxi, Hainan, Fujian; Vietnam (Tonkin).

#### Type material.

Holotype: male (10.2 mm long), Guangxi, Jinxiu, Shengtangshan, alt. 900 m, 1999.V.17, leg. Xingke Yang (IZAS, IOZ(E)1859448). Paratypes: **China:** **Guangxi:** 2 males, Jinxiu, Luoxiang, alt. 400 m, 1999.V.14, 15, leg. Decheng Yuan (IZAS, IOZ(E)1859449, 1859447); 1 male, Guangxi, Nanning, Wuming county, Mt. Damingshan [23°24'N, 108°28'E], alt. 1200 m, 2011.VII.11, coll. Yanquan Lu (CWD). **Hainan:** 1 female, Hainan Exp. 1934.IV.18 (IZAS, IOZ(E)1859445); 1 male, Hainan Exp. 1934.III.26 (IZAS, IOZ(E)1859446); 1 male 1 female, Hainan, Lingshui county, Diaoluoshan, alt. 1000 m, 2010.IV.23, leg. Ziwei Yin (SHEM); 1 female, Hainan, Ledong, Jiangfengling, Mingfenggu, 2011.V.25, alt. 1000 m, leg. Wenxuan Bi (CBWX); 1 female, Hainan, Ledong, 1962.IX.17, leg. Yaoquan Li (SYSU, En-366130); 1 male, Ledong, Jianfengling, Tianchi, 1948.VII.27, leg. Yi Liang (SYSU, En-366148). **Fujian:** 1 female, Wuyishan Nature Reserve, 2009.VII.10–17, leg. Ming Jin (CJM). **Vietnam:** 1 female, Tonkin, Backan, 1907, leg. Lemee (IZAS, ex MNHN, ex Coll. R. Oberthür, 1952, IOZ(E)1859450).

### Glenea lacteomaculata quadriguttata

Pic

http://species-id.net/wiki/Glenea_lacteomaculata_quadriguttata

Figs 25–26

Glenea 4-guttata  Pic, 1926: 22.
Glenea (Glenea) lacteomaculata  sbsp. *quadriguttata*; Breuning, 1956: 743.

#### Remarks.

Due to lack of material from Taiwan, the differences between *Glenea lacteomaculata* Schwarzer, 1925 and *Glenea lacteomaculata quadriguttata* Pic, 1926 are doubtful for the authors. The specimens from Guangxi and Yunnan provinces are conspecific to *Glenea lacteomaculata quadriguttata* and herein the new localities are reported.

#### Distribution.

China (**new country record**): Guangxi, Yunnan; Vietnam (Tonkin).

#### Type specimen examined.

Syntype (Fig. 25), 1 female, Vietnam, Tonkin, Djang (MNHN, ex Collection M. Pic).

**Figures 24–26.**
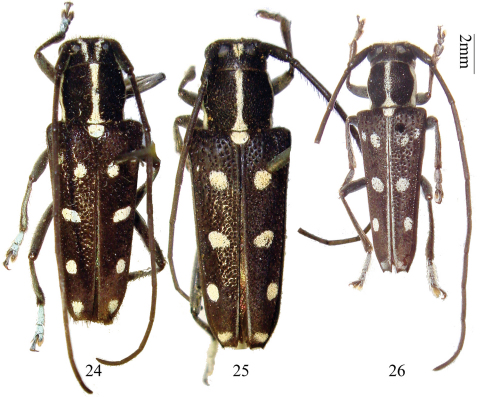
Habitus, *Glenea lacteomaculata* Schwarzer and *Glenea lacteomaculata quadriguttata* Pic. **24** paratype of *Glenea lacteomaculata*, female, from Taiwan, China. **25–26** *Glenea lacteomaculata quadriguttata* **25** syntype, female, from Tonkin, Vietnam **26** male, from Guangxi, China.

#### Other specimens examined.

**China: Guangxi:** 1 male (Fig. 26), Longzhou, Nonggang, alt. 240m, 1982.V.19, leg. Jikun Yang (CAU); 1 female, Guangxi Baohuqu, 1983.V, leg. Xiangtian Kong (IZAS); 2 females, Longrui, 1980.VI.1 (SYSU, En. 366147). **Yunnan:** 2 males, Hekou, Nanxi, Huayudong, alt. 150 m, 2010.IV.28, leg. Xiaodong Yang (CCCC). **Vietnam:** 5 males 4 females, Tonkin occ. Env. de Hoa-Binh, 1919, leg. R.P.A. de Cooman (MNHN, ex Coll. R. Oberthür, 1952); 1 male 1 female, Tonkin Env. De Hoa-Binh (MNHN, ex Coll. R. Oberthür, 1952); 2 females, Tonkin, HoaBinh, 1939.VII, leg. A. de Cooman (SYSU, Ce-002361–002362).

### Glenea subalcyone

Breuning

http://species-id.net/wiki/Glenea_subalcyone

Figs 28–29

Glenea  (*s. s*) *subalcyone* Breuning, 1964: 20, fig. page 21.
Glenea  (*s. str.*) *subalcyone*; Rondon and Breuning 1971: 535.

#### Remarks.

**Figures 27–29.**
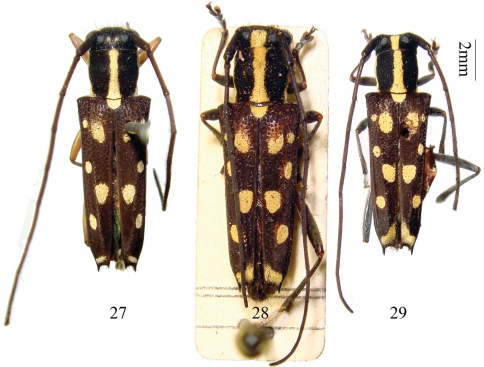
Habitus, *Glenea laodice* Thomson and *Glenea subalcyone* Breuning **27** holotype of *Glenea laodice*, female, from Laos. **28–29** *Glenea subalcyone* **28** holotype, female, from Laos. **29** female, from Yunnan, China.

This species is very similar to *Glenea laodice* Thomson, 1879 (Fig. 27), but can be distinguished by having legs black instead of testaceous. It is recorded from Chinese fauna for the first time.

#### Distribution.

China (**new country record**): Yunnan; Laos.

#### Type specimen examined.

Holotype (Fig. 28), female, Laos, région de Thakhek (Phontiou in label), 1963.VI, leg. J. A. Rondon (BM).

#### Other specimens examined.

**China: Yunnan:** 1 female (Fig. 29), Yiwubanna, Menglun, alt. 650 m, 1959.VIII.27, leg. Facai Zhang (IZAS).

### Glenea tonkinea

Aurivillius

http://species-id.net/wiki/Glenea_tonkinea

Figs 30–33

Glenea tonkinea  Aurivillius, 1925: 521, fig. 160 (Tonkin). [MNHN]
Glenea (Glenea) tonkinea  m. *basirufofemorata* Breuning, 1956: 743 (Tonkin). [MHNL]
Glenea (Glenea) tonkinea  m. *apicetruncata* Breuning, 1956: 743 (Tonkin). [NMB]

#### Diagnosis.

Differs from *Glenea pici* Aurivillius in having pubescent maculae white; in having different male claws. Differs from *Glenea lacteomaculata* Schwarzer in having spot at middle of elytron transverse, anterior claw in mid tarsus of male with long tooth (Fig. 31).

**Figures 30–32.**
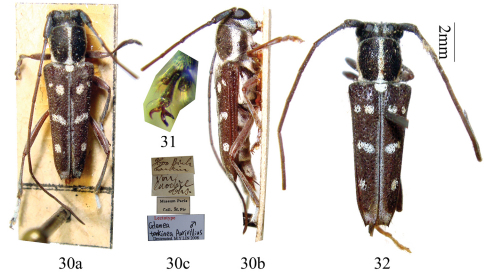
Habitus, *Glenea tonkinea* Aurivillius. **30** lectotype, male, from Tonkin,Vietnam **31** claw of mid tarsus in male (not to scale) **32** cotype of *Glenea tonkinea* m. *apicetruncata* Breuning, 1956, male, from Tonkin, Vietnam.

#### Lectotype designation.

**Figures 33–35.**
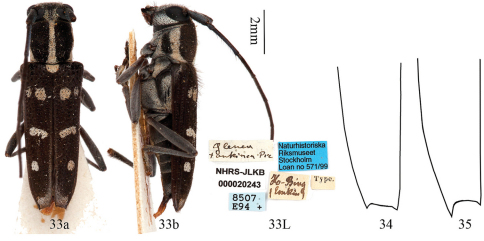
33 Habitus, *Glenea tonkinea* Aurivillius, paralectotype, female, from Tonkin, Vietnam. **a** dorsal view **b** lateral view. **L** labels. **34–35** elytral apex of left elytron (not to scale) **34** showing short teeth, tooth length at the outer angle subequal to that at the inner angle **35** showing long spine at the outer angle, much longer than that at the inner angle.

According to Aurivillius' original description, there were multiple type specimens, deposited in “Reichsmuseum in Stockholm und Collectio Pic". In order to fix the species concept and ensure universal and consistent interpretation of this species, we designate the male specimen in MNHN as the lectotype (Figs 30–31, 8.5 mm long, 2.4 mm wide) and the female in NHRS as the paralectotype (Fig. 33) of *Glenea tonkinea* Aurivillius.

#### Remarks.

The record from Taiwan is doubtful. It might be based on *Glenea diversenotata* Schwarzer, whose taxonomic position was not clear yet.

#### Distribution.

China **(**Taiwan?, Hainan, Guangxi); Vietnam (Tonkin), Myanmar (**new country record**).

#### Type specimens examined.

Lectotype, male, Tonkin, Hoo Binh (=Hoa Binh) (MNHN, ex Coll. M. Pic). Paralectotype, female, Tonkin, Ho Bing (=Hoa Binh) (NHRS-JLKB000020243). Holotype of *Glenea (Glenea) tonkinea* m. *apicetruncata* Breuning, male, Tonkin Mts. Mauson, alt. 2000–3000 feet, IV–V, leg. H. Fruhstorfer (NMB, ex Coll. Frey). Type of *Glenea (Glenea) tonkinea* m. *basirufofemorata* Breuning, male, Tonkin, Hoa Binh (MHNL, ex Coll. Lepesme); paratype, female, same data.

#### Other specimens examined.

**China: Guangxi:** 1 male, Longzhou, Nonggang, alt. 330 m, 2000.VI.5, leg. Wenzhu Li (IZAS); 1 male, Longzhou, Shida, 1980.V.24 (SYSU, En-366136); 1 female, Guangxi, Xiashi, 1963.V.7, leg. Jikun Yang (CAU). **Vietnam:** 1 female, Tonkin, Hoa-Binh (IRSNB); 3 males 3 females, same data but (MNHN, ex Coll. M. Pic); 1 male 1 female, Tonkin occ. Env. de Hoa-Binh, 1919, leg. A. de Cooman (MNHN, ex Coll. R. Oberthür, 1952); 1 female, Tonkin (MNHN, ex Coll. M. Pic); 1 male, Tonkin N. env. D'ha-Giang, 1914, leg. Lieut (MNHN); 1 female, Tonkin Reg. de Hao-Binh, 1927, leg. A. de Cooman (MNHN); 1 female, Tonkin, Baokan, 1907.VIII, leg. P. Lemee (MNHN, ex Coll. R. Oberthür, 1952); 1 female, Tonkin centr. Region de Chiem-Hoa et de Tuyen-Quam, 1901, leg. A. Weiss (MNHN). **Myanmar:** 1 female, Birmanie (Hte.) Mines des Rubis, alt. 1200–2300 m, 1890, leg. Doherty (MNHN).

## Discussion

The above species are grouped as *Glenea coomani* group by the following characters (not meant to be presumed synapomorphies, but rather only for identification of species having similar makings): pronotum largely black, generally with a white or yellow median stripe; body covered with pubescence instead of metallic squama (such as the metallic green maculae of *Glenea pici* Aurivillius, 1925); elytron black with an apical spot and 4 or 5 unequal sized spots. They differ from *Glenea relicta* group by elytral spots (not include the band just before apex) with unequal size and located in different position.

### Key to species of Glenea coomani group

**Table:** 

1	Elytral apex only having a short tooth at the outer angle (subequal to that at the inner angle, Fig. 34)	2
–	Elytral apex having a long and sharp spine at the outer angle (much longer than that at the inner angle, Fig. 35)	4
2	Elytron having only one big oval macula at basal fourth; the second macula located behind the basal one smallest (Figs 1–4)	*Glenea coomani*
–	Elytron having two small spots at basal fourth, the first spot is the smallest one	3
3	Elytral apex truncate or slightly emarginate, the middle spot on elytron almost rounded (Figs 25–26) or somewhat transverse (still far from suture, Fig. 24); male with apex of tergite VIII truncate	*Glenea lacteomaculata* (including the subspecies *Glenea lacteomaculata quadriguttata*)
–	Elytral apex obliquely truncate, the middle spot on elytron transverse and oblique (almost touching sutural stripe, Figs 30–33); male with apex of tergite VIII doubly emarginate (with a middle projection)	*Glenea tonkinea*
4	Elytron having 5 white or yellow maculae; legs black (Figs 12–15)	*Glenea neohumerosa* sp. n.
–	Elytron having 6 white or yellowbrown maculae	5
5	Legs testaceous; elytral apical spot smaller, not touching suture; vertex with two yellowbrown spots between upper eye lobes (Fig. 27)	*Glenea laodice*
–	Legs black; elytral apical spot bigger and touching suture; vertex with one yellowbrown spot between upper eye lobes (Figs 28–29)	*Glenea subalcyone*


## Supplementary Material

XML Treatment for Glenea coomani

XML Treatment for Glenea neohumerosa

XML Treatment for Glenea lacteomaculata quadriguttata

XML Treatment for Glenea subalcyone

XML Treatment for Glenea tonkinea
